# Standardized visual EEG features predict outcome in patients with acute consciousness impairment of various etiologies

**DOI:** 10.1186/s13054-020-03407-2

**Published:** 2020-12-07

**Authors:** Michael Müller, Andrea O. Rossetti, Rebekka Zimmermann, Vincent Alvarez, Stephan Rüegg, Matthias Haenggi, Werner J. Z’Graggen, Kaspar Schindler, Frédéric Zubler

**Affiliations:** 1 Department of Neurology, Sleep-Wake-Epilepsy-Center, Inselspital, Bern University Hospital, University of Bern, Bern, Switzerland; 2 Department of Clinical Neuroscience, Lausanne University Hospital (CHUV) and University of Lausanne, Lausanne, Switzerland; 3 Department of Neurology, Hôpital du Valais, Sion, Switzerland; 4 Department of Neurology, University Hospital Basel, Basel, Switzerland; 5 Department of Intensive Care Medicine, Inselspital, Bern University Hospital, University of Bern, Bern, Switzerland; 6 Department of Neurology, Inselspital, Bern University Hospital, University of Bern, Bern, Switzerland; 7 Department of Neurosurgery, Inselspital, Bern University Hospital, University of Bern, Bern, Switzerland

**Keywords:** Electroencephalography, Prognostication, Acute consciousness impairment, Hypoxic ischemic encephalopathy, Traumatic brain injury, Random forest

## Abstract

**Background:**

Early prognostication in patients with acute consciousness impairment is a challenging but essential task. Current prognostic guidelines vary with the underlying etiology. In particular, electroencephalography (EEG) is the most important paraclinical examination tool in patients with hypoxic ischemic encephalopathy (HIE), whereas it is not routinely used for outcome prediction in patients with traumatic brain injury (TBI).

**Method:**

Data from 364 critically ill patients with acute consciousness impairment (GCS ≤ 11 or FOUR ≤ 12) of various etiologies and without recent signs of seizures from a prospective randomized trial were retrospectively analyzed. Random forest classifiers were trained using 8 visual EEG features—first alone, then in combination with clinical features—to predict survival at 6 months or favorable functional outcome (defined as cerebral performance category 1–2).

**Results:**

The area under the ROC curve was 0.812 for predicting survival and 0.790 for predicting favorable outcome using EEG features. Adding clinical features did not improve the overall performance of the classifier (for survival: AUC = 0.806, *p* = 0.926; for favorable outcome: AUC = 0.777, *p* = 0.844). Survival could be predicted in all etiology groups: the AUC was 0.958 for patients with HIE, 0.955 for patients with TBI and other neurosurgical diagnoses, 0.697 for patients with metabolic, inflammatory or infectious causes for consciousness impairment and 0.695 for patients with stroke. Training the classifier separately on subgroups of patients with a given etiology (and thus using less training data) leads to poorer classification performance.

**Conclusions:**

While prognostication was best for patients with HIE and TBI, our study demonstrates that similar EEG criteria can be used in patients with various causes of consciousness impairment, and that the size of the training set is more important than homogeneity of ACI etiology.

## Background

Outcome prediction in patients with acute consciousness impairment (ACI) in the intensive care unit is essential in order to inform the relatives and avoid futile treatment [1, 2]. Prognostication is based on clinical and paraclinical examinations including blood tests, neuroimaging and electrophysiology [3]. The underlying cause of the ACI is considered to be relevant while trying to predict the clinical outcome, mainly for two reasons: firstly, because different etiologies are inherently associated with different mortality rates (typically higher for anoxic-ischemic encephalopathy (HIE) after cardiac arrest than for intoxication or status epilepticus, for instance [4]). Secondly, prognostication algorithms—and the relative importance of each modality—are different depending on the postulated etiology [3, 5]. This is especially true for electroencephalography (EEG). In patients with HIE, EEG has become the main prognostic tool, as several visual and quantitative (computer-derived) features have been shown to predict functional outcome [6–13]. In particular, a continuous and reactive EEG background suggests a favorable outcome, whereas a suppressed background or burst suppression with identical bursts is usually predictor of poor outcome [7, 8]. By contrast, EEG is not part of the main current prognostic scores used after traumatic brain injury (TBI), which instead rely on clinical markers, neuroimaging and blood values [14–16]. However, it has been known for decades that EEG can correlate with the severity of head injury [17, 18]. More recent studies demonstrated that visual EEG features such as background reactivity [19], continuity [20] or presence of stage N2 sleep transients [20] were associated with a favorable outcome after TBI. Using quantitative methods, background amplitude, frequency and variability were also shown to predict clinical outcome [21, 22]. The same holds true for subarachnoid hemorrhage: while EEG is not integrated into current prognostic tools [23], many features such as stable alpha rhythm [24], presence of sleep architecture or epileptiform activity [25, 26] can help predict the outcome.

The fact that the same EEG features (background reactivity, continuity, amplitude) have been used as prognostic markers in various etiologies raises the question whether similar criteria/decision making algorithms could be applied to a cohort of patients with various ACI etiologies. A few studies have applied visual or quantitative EEG criteria to prognostication in various origins of coma/ACI, but usually focused on a single of a few variables [27–30]. Here, we investigate the prognostic value of a model combining 8 major visual features from the Standard Critical Care Terminology from the American Clinical Neurophysiology Society (ACNS) [31] trained on a prospectively acquired cohort of patients with various etiologies of coma. The model is applied to patients with mixed etiologies, and then to subgroups of patients with specific subcategories of etiologies. Because it reflects the functioning of brain neurons, we postulate that EEG should be able to contribute to prognostication in all patients with ACI regardless of the underlying etiology.

## Methods

### Patients and EEG recordings

We performed a post hoc analysis of data prospectively acquired during the multicentric study CERTA (Continuous EEG Randomized Trial in Adults; NCT03129438). Details of the study have been published elsewhere [32, 33]. In short, patients > 18 with disorder of consciousness of any etiology (defined as GCS ≤ 11 or FOUR ≤ 12) hospitalized on the Intensive or Intermediate Care Units of four Swiss hospitals (Lausanne University Hospital (CHUV), Sion Hospital, Bern University Hospital (Inselspital), Basel University Hospital) for whom an EEG was requested for medical reasons were included. Exclusion criteria of the original study were clinical or electroencephalographic signs for seizures in the 36 h or status epilepticus in the 96 h preceding randomization, a palliative situation or documented refusal to participate to a clinical study. After inclusion, patients were randomized to undergo either continuous EEG monitoring for 30–48 h, or two standard EEGs (20–30 min) within the same timeframe. The CERTA study and the post hoc analysis of EEG data were approved by the local ethic commissions (Project-ID 2017-00268).

Video-EEGs were performed with a NicoletOne system (Viasys Neurocare, Madison WI, USA) using 21 or 23 electrodes placed according to the international 10:20-system. In neurosurgical patients, a reduced montage with 11 electrodes could be used [34]. For the present study, we considered only the first EEG (in case two standard EEGs were performed) or the first interpretation documented (in case of continuous EEG). EEG traces were interpreted during hospitalization by board-certified electroencephalographers with additional certification in ACNS Standard Critical Care Terminology [31] (AOR, RZ, VA, SR, KS, FZ).

### Etiology and outcome

At recruitment time, 14 non-mutually exclusive etiologies of ACI were registered. For the present study, we retrospectively formed four mutually exclusive etiologic groups, namely (1) *Stroke:* ischemic stroke, hemorrhagic stroke, non-traumatic subarachnoid hemorrhage; (2) *TBI*/*NS:* traumatic brain injury (TBI), subdural hematoma or other non-vascular non-traumatic neurosurgical conditions such as postoperative coma after brain tumor resection; (3) *MIII:* metabolic disturbance, intoxication, infection, inflammation; (4) *HIE:* hypoxic–ischemic encephalopathy after cardiac arrest from cardiac or non-cardiac origin. In cases where the patient had diagnoses belonging to two of the above categories, the "most severe" diagnosis would be considered (e.g. a patient with metabolic disturbance after HIE would be attributed to the etiologic group HIE). Two patients with both subarachnoid hemorrhage and HIE were not attributed to any diagnostic group (due to "equal severity"). The above-mentioned categorization was performed prior to data analysis. The rational for grouping traumatic and non-traumatic non-vascular neurosurgical diagnoses was the presence of brain lesions and possible skull defect (causing a breach rhythm in the EEG) in both categories. However, since outcome prediction in critically ill patients with traumatic brain injury is of particular interest, we also considered in the present study a subgroup with "pure" TBI patients (a subset of the TBI/NS subgroup described above).

As was the case in the CERTA study, we considered as primary outcome the mortality at 6 months, which was prospectively collected. We also considered a secondary outcome based on the best cerebral performance category (CPC) value reached within 6 months [35], dichotomized into a favorable (CPC 1 or 2) or unfavorable outcome (CPC 3–5).

### Electroencephalographic and clinical features

Electroencephalographic and clinical features were used to predict the outcome (Table 1). EEG features were based on the ACNS standard terminology [31] and consisted of: background continuity, background amplitude, background predominant frequency, background reactivity, background symmetry, presence of stage Non-REM 2 sleep transients, presence of sporadic (i.e. non-rhythmic or periodic) epileptiform discharges and finally presence of rhythmic or periodic patterns. Five demographic and clinical features were considered, namely age, gender, Glasgow coma scale (GCS) at inclusion time (just prior to EEG recording), C-reactive protein (CRP) value during the first EEG and etiology of coma postulated within 1 week after inclusion.

**Table 1:** Electroencephalographic (EEG) and clinical features used for prognostication

Name	Type	Categories/values
*EEG features*		
EEG background continuity	Ordinal	1. Continuous/nearly continuous
		2. Discontinuous
		3. Burst suppression
		4. Suppressed
Background amplitude	Ordinal	1. < 10 µV
		2. 10–20 µV
		3. > 20 µV
Background frequency	Ordinal	1. 1–3.5 Hz
		2. 4–7.5 Hz
		3. 8–13 Hz
		4. > 13 Hz
Background reactivity	Binary	Absent/present
Background symmetry	Binary	Absent/present
Stage II sleep transients	Binary	Absent/present
Sporadic epileptiform discharges	Binary	Absent/present
Rhythmic or periodic patterns ("main term 2")	Categorical	1. Periodic discharges
		2. Rhythmic delta activity
		3. Rhythmic spike waves/sharp wave
		4. No rhythmic or periodic pattern
*Clinical features*		
Age	Continuous	Real number
Gender	Binary	Female/male
Glasgow coma scale	Ordinal	3–15
C-reactive protein	Continuous	Real number
Etiology	Categorical	1. Stroke
		2. Trauma/neurosurgery
		3. Toxic/metabolic/infectious
		4. Hypoxic ischemic encephalopathy
		5. No etiology available

### Classification model

We used random forest (RF) classifiers [36] to predict clinical outcome. A RF consists of multiple decision trees that have been devised successively using the training set. For classification, the decision of all the trees is aggregated to reach a global decision. We chose RF because of their natural ability to handle all types of variables, continuous, ordinal and nominal, without any preceding reduction or transformation of the predictor space. In addition, RF models have a relatively low risk of overfitting, and they allow to easily counteract unbalanced data sets by setting class weights [37]. Finally, it has been shown that good performances are achieved without hyperparameter tuning (that is, using standard parameters) [38]. RF also provides a way to estimate the relative importance of the respective features (by evaluating their role as splitting variables in the decision trees composing the random forest), even though a direct computation of the predictive value (such as odds ratios) of each feature is not possible.

We first considered all patients, regardless of the underlying ACI etiology ("general classifier"). For this task, patients were divided into a stratified training set (2/3 of patients, *N* = 242) and a test set (1/3, *N* = 122). Each classifier was sequentially trained using EEG features alone, clinical features alone and finally a combination of EEG and clinical features.

We then trained RF classifiers independently on each etiology subgroup ("specific classifiers"). Because of the limited number of subjects in each subgroup, we omitted a separate test set and used instead fivefold cross-validation on all available data.

For each classification task, 500 trees with a maximal depth of $\left\lfloor {m/2} \right\rfloor$, where *m* is the number of incorporated features, were trained using the AdaBoost algorithm [39] with a learning rate of 0.1. Because RF, as most classification algorithms, tends to be biased toward the majority class, we used a weighted approach to penalize misclassifying the minority class [40], whereby class weights were 1/relative frequencies. All parameters were defined prior to analysis and were not optimized on data.

We assessed the performance of the RF classifiers with areas under the receiving operating characteristics (ROC) curve (AUC). The 95% confidence intervals of ROC curves and corresponding AUC values were determined via 3000 bootstrapped replicas. For the general classifier, we also computed accuracy, sensitivity, specificity, positive predictive value and negative predictive value with their binomial 95% confidence intervals. Statistical significance of differences between AUCs was assessed with a Z test [41]

RF classifiers were implemented in MATLAB version 2015b (MathWorks) using the function *fitensemble* from the *Statistics and Machine Learning* Toolbox. Unless stated otherwise, parameters were set to their default values.

## Results

### Patients

A total of 364 patients (34% women, mean age 64 ± 15 years) for whom the outcome at 6 month was known were included, of which 187 (51%) survived, and 139 (38%) had a favorable outcome (for details about the inclusion and drop-out see [32]). The detailed number of patients in each etiological category is shown in Table 2.

**Table 2:** Etiology and outcome distribution

Etiology	*N*	Age [IQR]	Female (%)	Survival (%)	Favorable outcome (%)	EEG delay [IQR]
Stroke	82	67 [55 78]	41 (50.0)	37 (45.1)	21 (25.6)	69 [37 122]
TBI/NS	50	63 [40 73]	13 (26.0)	33 (66.0)	24 (48.0)	75 [43 115]
TBI	44	63 [40 73]	12 (27.3)	30 (68.2)	21 (47.7)	71 [43 112]
MIII	47	62 [51 73]	14 (29.8)	26 (55.3)	22 (46.8)	139 [48 268]
HIE	110	66 [54 75]	30 (27.3)	42 (38.2)	34 (30.9)	24 [17 52]
No etiology available at recruitment time	75	68 [59 77]	25 (33.3)	49 (65.3)	38 (50.7)	101 [33 192]
All	364	67 [55 75]	123 (33.8)	187 (51.4)	139 (38.2)	59 [24 138]

*EEG delay* time between admission and EEG (in hours), *HIE* hypoxic–ischemic encephalopathy, *MIII* metabolic, intoxication, infection, inflammation, *TBI* traumatic brain injury, *TBI/NS* traumatic brain injury and other non-traumatic non-vascular neurosurgical diagnosis

### Performance of the general classifiers

**Fig. 1:**
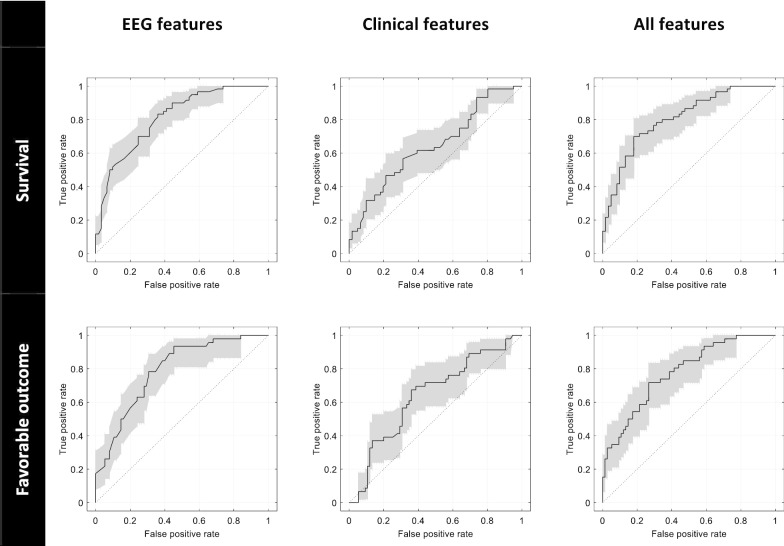
Receiver operating characteristic curves (black) with 95% confidence intervals (gray) of the general models obtained on the test set (121 patients)

The detailed performances of the general classifiers (trained on patients with all etiologies) are presented in Table 3, whereas ROC curves are shown in Fig. 1. Using EEG features, the area under the ROC curve was 0.812 for predicting survival and 0.790 for predicting favorable outcome. The prediction using the EEG features alone was more accurate than with clinical features alone for both outcomes (*p* = 0.008 for survival, *p* = 0.031 for favorable outcome). A combination of both sets of features did not improve the performance compared to EEG features alone (*p* = 0.984 for survival, *p* = 0.887 for favorable outcome).

**Table 3:** Performance of the general classifiers for predicting survival at 6 months or for predicting a favorable outcome (CPC 1 or 2) using different features. Point estimates and 95% confidence intervals

	AUC	Accuracy	Sensitivity	Specificity	PPV	NPV
*Predicting survival*						
EEG features	.812	.736	.833	.639	.694	.796
	[.721 .874]	[.657 .814]	[.739 .928]	[.519 .760]	[.588 .801]	[.683 .909]
Clinical features	.643	.596	.617	.574	.587	.603
	[.535 .737]	[.508 .683]	[.494 .740]	[.450 .698]	[.466 .709]	[.478 .729]
All features	.806	.752	.717	.787	.768	.739
	[.721 .871]	[.675 .829]	[.603 .831]	[.684 .890]	[.657 .878]	[.632 .845]
*Predicting*						
*Favorable outcome*						
EEG features	.790	.703	.848	.613	.574	.868
	[.693 .862]	[.621 .784]	[.744 .952]	[.503 .724]	[.456 .691]	[.777 .959]
Clinical features	.641	.628	.587	.653	.509	.721
	[.537 .736]	[.542 .714]	[.445 .729]	[.546 .761]	[.375 .644]	[.614 .827]
All features	.777	.703	.565	.787	.619	.747
	[.687 .852]	[.621 .784]	[.422 .709]	[.694 .879]	[.472 .766]	[.651 .843]

Our relatively large test set allowed for a subanalysis of the performance of the general classifier depending on the etiology, whereby only EEG features were used (Table 4). The prediction performance was better for the subgroups of patients with TBI/NS (including the subgroup with pure TBI) or HIE than for patients with stroke or with metabolic, inflammatory, infectious origin of consciousness impairment. The between group difference of AUC values was statistically significant for predicting survival (*p* < 0.05 for HIE-Stroke, HIE-MIII, TBI/NS-Stroke, TBI/NS-MIII).

**Table 4:** Performance of the general classifier (that is, trained on patients with all etiologies) and of the specific classifiers (independently trained on subgroups of specific etiology) for predicting outcome in subgroup of different etiologies using EEG features. AUC and 95% confidence intervals

	Stroke	TBI/NS	TBI	MIII	HIE	No etiology
*Predicting survival*						
General classifier	.695	.955	.944	.697	.958	.737
	[.433 .878]	[.734 1.000]	[.628 1.000]	[.423 .894]	[.827 .996]	[.442 .936]
Specific classifiers	.666	.694	.800	.452	.833	.646
	[.534 .769]	[.469 .851]	[.571 .907]	[.290 .628]	[.741 .902]	[.488 .775]
*Predicting*						
*Favorable outcome*						
General classifier	.706	.819	.866	.742	.921	.678
	[.474 .880]	[.472 .972]	[.500 1.000]	[.475 .909]	[.762 .979]	[.404 .876]
Specific classifiers	.662	.651	.723	.396	.865	.652
	[.510 .776]	[.480 .803]	[.547 .851]	[.240 .568]	[.781 .923]	[.512 .765]

*HIE* hypoxic–ischemic encephalopathy,* MIII* metabolic, intoxication, infection, inflammation,* TBI* traumatic brain injury,* TBI/NS* traumatic brain injury combined with other non-vascular non-traumatic neurosurgical diagnoses

**Fig. 2:**
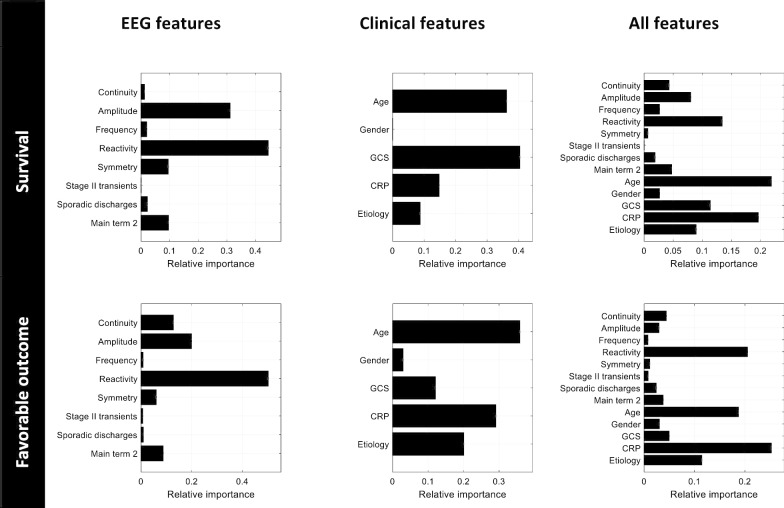
Relative importance of features in the general models (that is, after training on patients with all etiologies) for three different feature sets and two different outcomes. GCS: Glasgow coma scale; CRP: C-reactive protein; Main term 2: presence of rhythmic or periodic patterns

Figure 2 illustrates the relative importance of the different features in the general classifiers. EEG background reactivity was the most important electroencephalographic feature for both outcomes. The most important clinical features were Glasgow coma score (closely followed by age) for predicting survival, and the age for predicting a favorable vs. unfavorable outcome. Of note, reactivity remained the main EEG feature when both electroencephalographic and clinical features were used.

### Performance of the specific classifiers

Specific classifiers were then trained independently on subgroups of patients with related etiologic category based on EEG features alone and using fivefold cross-validation. The performances of the specific classifiers were systematically lower than those of the general classifier on patients from the same etiology (Table 4). The drop in performance was statistically significant for the TBI/NS and HIE subgroups for predicting survival (*p* = 0.004 and *p* = 0.037, respectively) and for the MIII subgroup for predicting a favorable outcome (*p* = 0.010).

**Fig. 3:**
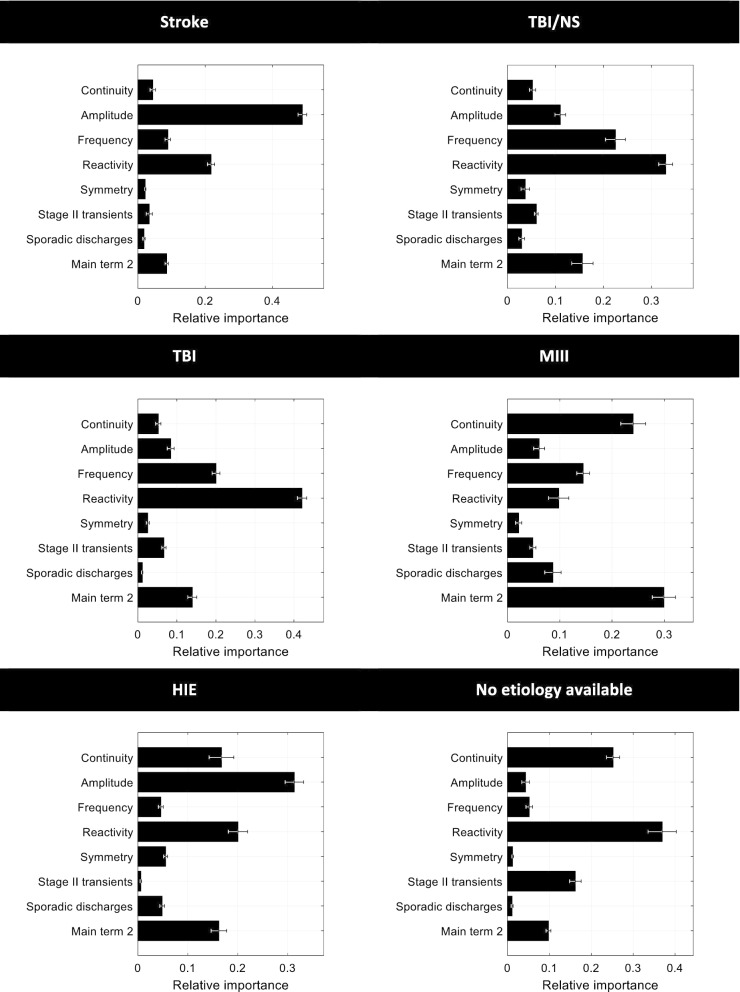
Relative importance of features in the specific models (each trained on a specific etiology group) for predicting survival. Bars represent the mean; error bars represent the standard error of the mean for the 5 models trained during cross-validation. Main term 2: presence of rhythmic or periodic patterns. *HIE* hypoxic–ischemic encephalopathy, *MIII* metabolic, intoxication, infection, inflammation, *TBI* traumatic brain injury, *TBI/NS* traumatic brain injury combined with other non-vascular non-traumatic neurosurgical diagnoses

**Fig. 4:**
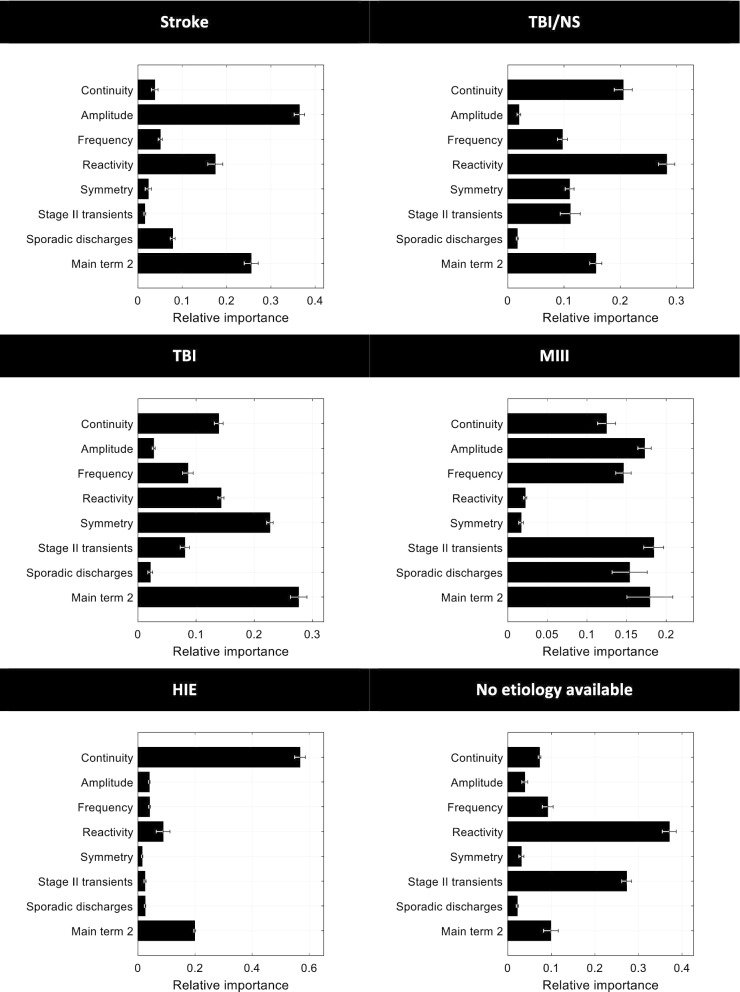
Relative importance of features in the specific models (that is, trained on a specific etiology group) for predicting favorable outcome. Bars represent the mean; error bars represent the standard error of the mean for the 5 models trained during cross-validation. Main term 2: presence of rhythmic or periodic patterns. Abbreviations as in Fig. 3

The relative importance of the single EEG features in the different etiological groups is presented in Fig. 3 for predicting survival and in Fig. 4 for predicting favorable outcome. Of note, EEG background reactivity was the most important feature only in the subgroups of TBI/neurosurgery patients, as well as in the subgroup of patients for which the etiology was not known.

## Discussion

We used electroencephalographic and clinical features to predict the outcome in critically ill patients with acute consciousness impairment using a random forest classifier. The main result of our study is that despite training the classifier on a cohort of patients with different ACI etiologies, an AUC of 0.812 for predicting survival based on EEG features was achieved.

So far, only a minority of studies on EEG-based prognostication were conducted on patients with different etiologies of coma or ACI. EEG background reactivity, for instance, has been shown repetitively to be an important predictor in almost all groups of patients (HIE, TBI, toxic-metabolic; for reviews see [19, 42]). Several pre-defined EEG patterns were used to predict outcome in patients with global (HIE) and focal (hemispheric infarct) ischemic lesions [27]. A combination of EEG reactivity and presence of sleep spindles predicted 1-month awakening in patients in coma for at least 3 days [28]. Presence of at least a unilateral posterior dominant rhythm was associated with survival in patients admitted to a neurologic intensive care unit for various diagnoses [43]. Also quantitative analysis has been applied to patients with various etiology of coma: a combination of different synchronization measures was shown to predict survival at discharge from the ICU [29]; amplitude integrated EEG applied during 6–12 h one week after brain injury (including TBI and stroke) onset could successfully predict functional outcome [30]. Taken together, these and our results seem to indicate that similar EEG criteria can be applied for outcome prediction regardless of the etiology of ACI/coma.

The performance of the classifier trained on all etiologies was especially good in the subgroup of patients with HIE, which was expected, but also in patients with TBI (isolated, or grouped with patients with other non-traumatic non-vascular neurosurgical diagnoses). Our results are in line with previous studies showing that EEG has a potential role in prognostication in TBI [18, 18], despite the fact that EEG is not routinely used as a prognostic tool in this condition [3]. In comparison, the performance was lower in the subgroups of patients with toxic, metabolic, inflammatory or infections origin. This group contains the largest proportion of patients with non-primary cerebral disorders; this heterogeneity might explain why a prognostic tool based on electric brain activity does not perform well. It is also known that relatively moderate metabolic encephalopathy can dramatically modify the EEG, and that several EEG patterns usually associated with a poor outcome, such as burst suppression or suppressed background, can be found even in reversible metabolic encephalopathies [44]. The performance was also lower in case of stroke. This could be due to the features chosen for this study, as only the presence, but not the severity of an asymmetry, was considered. Previous studies have shown that the degree of asymmetry is an important predictor for the functional outcome after stroke [45, 46].

Using clinical and demographic variables in addition to EEG features did not change the overall classification performance (AUC 0.812 vs. 0.806). However, this increased the specificity and decreased the sensitivity for predicting survival or a favorable outcome (Table 3). One can postulate that a "good" EEG is a necessary but not sufficient condition for guaranteeing a good outcome, and that ruling out other known predictors of poor outcome (such as a higher age) helps increasing the specificity for survival.

### General versus specific classifiers

Training the classifiers on subgroups of patients with similar etiologies led to poorer performances. However, it is important to note that in the present study, using more homogeneous subgroups also meant using less data: the training set for the general classifiers comprised 242 patients (1/3 of all patients), whereas for the specific classifiers the size of the training set was between 38 (for MIII) and 88 (for HIE) for each cross-validation run (4/5 of patients with a given etiology). It is known that a smaller training set increases the risk of overfitting and leads to poorer performance [47]. Overfitting could explain part of the large variation in relative feature importance observed when changing the outcome and etiologies in the specific classifiers (Figs. 3, 4**)**, whereas the unique combination of features from the general model was better for practically all subgroups. Our results suggest thus that using a larger training set, even at the cost of reducing the homogeneity of the group, can ultimately increase the performance of an EEG-based classifier. This is an important observation in an era where computer-assisted medicine is growing, but available data are often insufficient for modern machine learning methods—due to the cost of data labeling, and because data sharing between organizations is limited in practice [48]. Whether the same principle applies to other EEG-based clinical settings (such as the detection of epileptiform activity in various types of epilepsies, for instance) remains to be confirmed.

### Importance of EEG reactivity

When considering all patients together, EEG background reactivity (EEG-R) was the most important EEG feature, both for predicting survival and favorable functional outcome. EEG-R has been extensively used for prognostication in several etiologies [19, 42] and is thought to require functioning spino-thalamic and thalamo-cortical afferences [42]; it has been shown to correlate with neuron-specific enolase (a biomarker for neural lesion) [49] and topography of MRI lesions [50] in patients with HIE. Interestingly, EEG-R was no longer the most important feature when classifiers were trained specifically on subgroups of patients with similar etiology, except for the TBI/NS group (for which we can postulate that a known marker for the integrity of afferent pathways is a good predictor). Reactivity was also the most important feature in the subgroup of patients for whom the diagnosis was unknown at the time of the EEG, possibly due to the fact that this group was probably also very heterogeneous.

It might seem surprising that reactivity was not the most important feature for HIE, since it has been shown in numerous studies to be a good predictor for this condition. This can be due to the correlation of EEG-R with amplitude and continuity (both important features in this subgroup): since a suppressed background is usually not reactive, EEG-R is unlikely to be chosen as splitting variable in a sub-branch of a decision tree under a splitting node describing a flat line. We also note the importance of periodic or rhythmic pattern (Main term 2). Usually, the appearance or modification of periodic pattern or rhythmic spike waves following stimulus [51] is not considered background reactivity and can complicate the detection of true background reactivity. In summary, EEG-R could appear as being less important than it would if used in isolation due to its correlation with other variables and how this affects the RF algorithm.

EEG-R was not one of the most relevant features for the MIII subgroup. As already mentioned, the MIII group contains a larger proportion of patients with non-primarily cerebral cause for consciousness impairment. For these patients, probing the integrity of afferent pathways is less informative, in particular considering the fact that reactivity is often present, and thus less discriminative.

### Strengths and limitations

EEG and clinical data have been prospectively acquired in the course of a large multicentric study. EEG feature scoring has been performed by experienced encephalographers with by additional certification in ACNS terminology, which has been validated and offers good interrater agreement [52]. However, our study has several limitations. First, the decision to perform an EEG was taken by treating physicians based on clinical criteria. As such, there is a selection bias, since patients for whom a decision to withdraw life supporting therapy was already made, or patients who were quickly improving, were not included. Also, the timing of EEG after onset of consciousness impairment was not consistent between all patients. However, patient selection and timing of EEG correspond to "real-world" situations where an EEG is considered relevant by the treating physician. Of note, most HIE patients from the hospitals in Lausanne, Bern and Sion were recorded early after cardiac arrest (during targeted temperature management), since they were also included in an observational registry [6]. It is difficult to estimate how these selection biases influence the performance of the classifiers. It is possible, for instance, that a model trained on more systematically acquired data would lead to better results. By design of the CERTA study, no patient with known seizures in the previous 36 h or status epilepticus in the previous 90 h was included, which constitutes another selection bias. This bias is the reason why we did not include the presence of an electroencephalographic seizure as feature in our models. In case of known seizures, however, the response to anti-seizure medication is the main prognostic feature, and not the other ACNS criteria. As previously published [32], about 10% of patients in the present cohort (4.4% in those undergoing routine EEG, 15.7% in the group with continuous EEG) had ictal or interictal epileptiform activity during at least one EEG recording; while these patients were not excluded from the analysis, these numbers correspond to a recent meta-analysis of available cohort studies [53] and thus in our view reinforce generalizability of our findings. Also, we did not account for potential preexisting disability, which could affect the best CPC.

Self-fulfilling prophecy is a potential risk in all prognostication studies, especially in retrospective studies in which the decision for withdrawal of life supporting treatment (WLST) was left to the treating physician and not explicitly documented. However, EEG plays a decisive role in WLST decision mainly in patients with HIE. The majority of patients with HIE were recruited in Lausanne and Bern, where according to current guidelines, the first EEG (the one used for analysis in the present study) is usually not considered for decision to WLST [6, 54]. In patients with other etiologies, EEG is not a primary tool for clinical prognostication; therefore, the risk of self-fulfilling prophecy is probably limited.

Finally, the choice of clinical features was limited by the data registered during the CERTA study. Due to the lack of neuroradiological data, the complementarity of neuroradiological and electroencephalographic features could not be investigated. Also, we decided to consider a single blood test; the results might have been better with more blood markers, or after selection of the clinical values with a univariate or multivariate assessment. Of note, CRP levels have been shown to be correlated with the outcome in several etiologies, in particular in sepsis [55] and intracerebral hemorrhage [56].

## Conclusion

Currently, the role of EEG as a prognostic tool for critically ill patients with consciousness impairment strongly diverges based on the underlying etiology (major role in HIE, limited in TBI). In the same way that MRI and computer-tomography are now progressively incorporated into decision-making in HIE [50, 57, 58], this and other studies support the fundamental role of EEG as a prognostic tool for patients with TBI and possibly other etiologies. Further studies are needed to confirm the value of EEG and providing scoring system applicable in practice in patients with non-hypoxic etiology of coma.

## Data Availability

The dataset analyzed during the current study is not publicly available due to limitations from the local ethics committees but is available from the corresponding author on reasonable request.

## Abbreviations

ACI: Acute consciousness impairment
ACNS: American Clinical Neurophysiology Society
AUC: Area under the receiver operating characteristic (ROC) curve
CERTA: Continuous EEG randomized trial in adults
CPC: Cerebral performance category
CRP: C-reactive protein
EEG: Electroencephalography
EEG-R: EEG background reactivity
FOUR: Full outline of unresponsiveness (score)
GCS: Glasgow coma scale
HIE: Hypoxic ischemic encephalopathy
MIII: Metabolic disturbance, intoxication, infection, inflammation
RF: Random forest
TBI: Traumatic brain injury
TBI/NS: Traumatic brain injury combined with other non-vascular and non-traumatic neurosurgical diagnosis
WLST: Withdrawal of life supporting therapy
